# Poly[bis­[μ-1,3-bis­(diphenyl­phosphan­yl)propane-κ^2^ *P*:*P*′]-di-μ-thio­cyanato-κ^2^ *S*:*N*;κ^2^ *N*:*S*-disilver(I)]

**DOI:** 10.1107/S1600536811041250

**Published:** 2011-10-12

**Authors:** Li-Na Cui, Yu-Han Jiang, Li-Li Zhou, Qiong-Hua Jin, Cun-Lin Zhang

**Affiliations:** a Department of Chemistry, Capital Normal University, Beijing 100048, People’s Republic of China; b Research Center for Import–Export Chemicals Safety of the General Administration of Quality Supervision, Inspection and Quarantine of the People’s Republic of China (AQSIQ), Beijing 100123, People’s Republic of China; c Key Laboratory of Terahertz Optoelectronics, Ministry of Education, Department of Physics, Capital Normal University, Beijing 100048, People’s Republic of China

## Abstract

In the title coordination polymer, [Ag_2_(NCS)_2_(C_27_H_26_P_2_)_2_]_*n*_, two centrosymmetrically related Ag^+^ cations are linked by two thio­cyanate anions into binuclear eight-membered macrocycles. The Ag⋯Ag separation within the macrocycle is 5.4400 (6) Å. The distorted tetra­hedral coordination about each metal atom is completed by the P atoms of two bridging 1,3-bis­(diphenyl­phosphan­yl)propane ligands, forming polymeric ribbons parallel to the *a* axis.

## Related literature

For silver(I) complexes containing phosphane ligands and coordinated anions, see: Jin, Hu *et al.* (2010 ▶); Jin, Song *et al.* (2010 ▶); Effendy *et al.* (2007 ▶). For related structures, see: Cui, Hu *et al.* (2010 ▶); Cui, Jin *et al.* (2010 ▶); Mu *et al.* (2010 ▶); Affandi *et al.* (1997 ▶). 
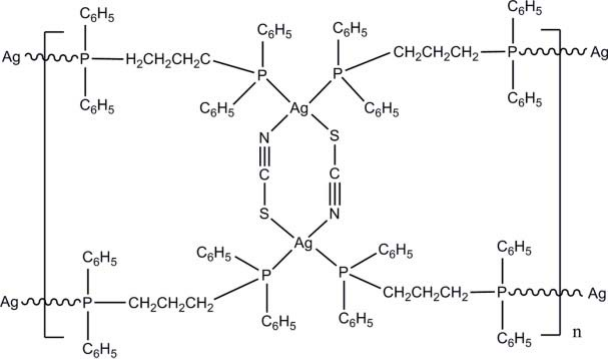


## Experimental

### 

#### Crystal data

[Ag_2_(NCS)_2_(C_27_H_26_P_2_)_2_]
*M* *_r_* = 1156.74
Monoclinic, 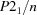

*a* = 7.5478 (9) Å
*b* = 15.8275 (17) Å
*c* = 22.229 (3) Å
β = 99.727 (1)°
*V* = 2617.4 (5) Å^3^
*Z* = 2
Mo *K*α radiation
μ = 0.99 mm^−1^
*T* = 298 K
0.42 × 0.21 × 0.15 mm

#### Data collection

Bruker SMART CCD area-detector diffractometer
Absorption correction: multi-scan (*SADABS*; Bruker, 2007 ▶) *T*_min_ = 0.682, *T*_max_ = 0.866
12975 measured reflections
4605 independent reflections
3068 reflections with *I* > 2σ(*I*)
*R*_int_ = 0.042

#### Refinement

*R*[*F* ^2^ > 2σ(*F* ^2^)] = 0.038
*wR*(*F* ^2^) = 0.079
*S* = 1.03
4605 reflections
298 parameters
H-atom parameters constrained
Δρ_max_ = 0.44 e Å^−3^
Δρ_min_ = −0.50 e Å^−3^

### 

Data collection: *SMART* (Bruker, 2007 ▶); cell refinement: *SAINT-Plus* (Bruker, 2007 ▶); data reduction: *SAINT-Plus*; program(s) used to solve structure: *SHELXS97* (Sheldrick, 2008 ▶); program(s) used to refine structure: *SHELXL97* (Sheldrick, 2008 ▶); molecular graphics: *SHELXTL* (Sheldrick, 2008 ▶); software used to prepare material for publication: *SHELXTL*.

## Supplementary Material

Crystal structure: contains datablock(s) global, I. DOI: 10.1107/S1600536811041250/rz2645sup1.cif

Structure factors: contains datablock(s) I. DOI: 10.1107/S1600536811041250/rz2645Isup2.hkl

Additional supplementary materials:  crystallographic information; 3D view; checkCIF report

## Figures and Tables

**Fig. 1.**
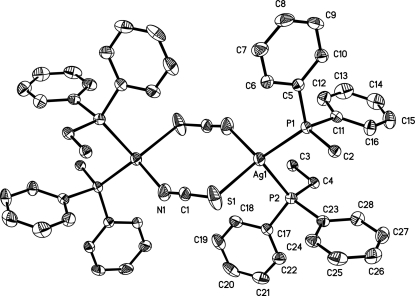
The structure of the basic unit of the title complex, with displacement ellipsoids drawn at the 30% probability level. Hydrogen atoms are omitted for clarity. Unlabelled atoms are related to the labelled atoms by the symmetry operation 1-x, 1-y, 1-z.

**Fig. 2.**
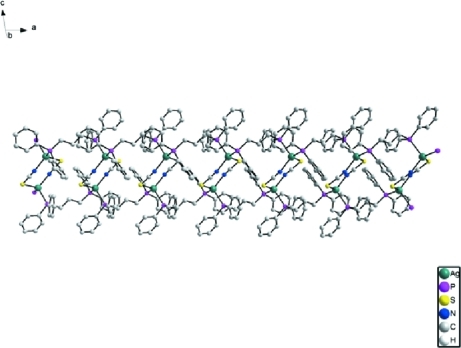
A view of the polymeric ribbon of the title compound running parallel to the a axis. Hydrogen atoms are omitted for clarity.

## supplementary crystallographic information

### Comment

Reports on the structural and kinetic features of silver(I) phosphane-oligodentate N-bases complexes are growing in number as the participation of these compounds in biological process and lunminescence materials are discovered (Jin, Hu *et al.*, 2010; Jin, Song *et al.*, 2010; Effendy *et al.*, 2007). We have studied the catalytic function of some nitrogen heterocyclic ligands, and found that some of them play an important role in the formation of products of mixed P and N-ligands with special structures. For examples, [Ag_4_(SCN)_4_dppm_2_] and [AgSCN(dppm)]_2_ (dppm = bis(diphenylphosphanyl)methane) were obtained under the catalysis of quinoline and phenanthroline, respectively. [AgClO_4_(PPh_3_)_3_] (Cui, Hu *et al.*, 2010), [AgClO_4_(PPh_3_)_3_(MeOH)] (Cui, Jin *et al.*, 2010) and [Ag(PPh_3_)(CH_3_COO)]_2_.H_2_O.CH_3_OH (Mu *et al.*, 2010) were prepared under the catalysis of 2-aminopyrimidine. Here we report the crystal structure of a new complex {[Ag(dppp)SCN]_2_}_n_ (dppp = bis(diphenylphosphanyl)propane) prepared under the catalysis of phenanthroline (phen).

The molecular structure of the title complex is depicted in Fig. 1. The polymeric complex can be described as originating from the repetition of a building block consisting of two Ag^+^ cations, two thiocyanate ions and two bis(diphenylphosphanyl)propane ligands. The coordination modes of dppp and SCN^-^ anion in 1 are different from those observed in complex [Ag(dppp)_2_]SCN.1.5py (py = pyridine) (2; Affandi *et al.*, 1997). In 1, both dppp ligands and SCN^-^ anions adopt a bridging mode, while in complex 2 the dppp ligands act as chelate ligands and the SCN^-^ anions act as free anions. In 1, each Ag atom is coordinated by two P atoms from two bridging dppp ligands, one S atom and one N atom from two SCN^-^ anions. Each thiocyanate ion bridges two centrosymmetrically related Ag^+^ ions in µ_2_-mode through the S and N atoms to form binuclear eight-membered macrocycles, the dppp ligand bridges two silver ions along the *a* axis through two P atoms to generate one-dimensional polymeric ribbons (Fig. 2). In 1, the AgP_2_SN coordination geometries could be described as distorted tetrahedral. The P1—Ag—P2, P1—Ag—S1, N1—Ag—S1 and P2—Ag—N1 angles are 102.18 (4)°, 122.60 (4)°, 103.26 (10)° and 109.61 (12)°, respectively. The P1—Ag—P2 angle is smaller than those in complex 2 (108.8 (7) and 120.3 (7)°) The Ag—P distances (2.5316 (11) and 2.4499 (10) Å) are similar to the average Ag—P distance in complex *2* (mean value 2.52 (2)Å).

### Experimental

The title complex has been prepared by adding phenanthroline (0.2 mmol, 0.0396 g) into a stirred mixture of of DMF (2 ml), CH_3_CN (5 ml) and MeOH (5 ml) containing AgSCN (0.2 mmol, 0.0332 g) and bis(diphenylphosphanyl)propane (0.2 mmol, 0.0825 g). Stirring continued for 3 h. After slow evaporation of the filtrate at ambient temperature for several days, white strip shaped crystals suitable for X-ray diffraction were obtained. Analysis found: C 49.60%, H 4.28%, N 11.51%; calculated: C 49.56%, H 4.33%, N 11.57%.

### Refinement

All hydrogen atoms were located in the calculated sites and included in the final refinement using the riding model approximation, with C—H = 0.93–0.97 Å, and with *U*_iso_(H) = 1.2 *U*_eq_(C).

### Crystal data

**Table:**

[Ag_2_(NCS)_2_(C_27_H_26_P_2_)_2_]	*F*(000) = 1176
*M**_r_* = 1156.74	*D*_x_ = 1.468 Mg m^−^^3^
Monoclinic, *P*2_1_/*n*	Mo *K*α radiation, λ = 0.71073 Å
Hall symbol: -P 2yn	Cell parameters from 3146 reflections
*a* = 7.5478 (9) Å	θ = 2.3–23.9°
*b* = 15.8275 (17) Å	µ = 0.99 mm^−^^1^
*c* = 22.229 (3) Å	*T* = 298 K
β = 99.727 (1)°	Prism, white
*V* = 2617.4 (5) Å^3^	0.42 × 0.21 × 0.15 mm
*Z* = 2	

### Data collection

**Table:**

Bruker SMART CCD area-detector diffractometer	4605 independent reflections
Radiation source: fine-focus sealed tube	3068 reflections with *I* > 2σ(*I*)
graphite	*R*_int_ = 0.042
φ and ω scans	θ_max_ = 25.0°, θ_min_ = 2.3°
Absorption correction: multi-scan (*SADABS*; Bruker, 2007)	*h* = −8→8
*T*_min_ = 0.682, *T*_max_ = 0.866	*k* = −18→18
12975 measured reflections	*l* = −26→23

### Refinement

**Table:**

Refinement on *F*^2^	Primary atom site location: structure-invariant direct methods
Least-squares matrix: full	Secondary atom site location: difference Fourier map
*R*[*F*^2^ > 2σ(*F*^2^)] = 0.038	Hydrogen site location: inferred from neighbouring sites
*wR*(*F*^2^) = 0.079	H-atom parameters constrained
*S* = 1.03	*w* = 1/[σ^2^(*F*_o_^2^) + (0.0218*P*)^2^ + 2.1307*P*] where *P* = (*F*_o_^2^ + 2*F*_c_^2^)/3
4605 reflections	(Δ/σ)_max_ = 0.002
298 parameters	Δρ_max_ = 0.44 e Å^−^^3^
0 restraints	Δρ_min_ = −0.50 e Å^−^^3^

### Special details

**Table:**

Geometry. All e.s.d.'s (except the e.s.d. in the dihedral angle between two l.s. planes) are estimated using the full covariance matrix. The cell e.s.d.'s are taken into account individually in the estimation of e.s.d.'s in distances, angles and torsion angles; correlations between e.s.d.'s in cell parameters are only used when they are defined by crystal symmetry. An approximate (isotropic) treatment of cell e.s.d.'s is used for estimating e.s.d.'s involving l.s. planes.
Refinement. Refinement of *F*^2^ against ALL reflections. The weighted *R*-factor *wR* and goodness of fit *S* are based on *F*^2^, conventional *R*-factors *R* are based on *F*, with *F* set to zero for negative *F*^2^. The threshold expression of *F*^2^ > σ(*F*^2^) is used only for calculating *R*-factors(gt) *etc*. and is not relevant to the choice of reflections for refinement. *R*-factors based on *F*^2^ are statistically about twice as large as those based on *F*, and *R*- factors based on ALL data will be even larger.

### Fractional atomic coordinates and isotropic or equivalent isotropic displacement parameters (Å^2^)

**Table:**

	*x*	*y*	*z*	*U*_iso_*/*U*_eq_	
Ag1	0.67725 (4)	0.369866 (19)	0.563731 (15)	0.04506 (12)	
P1	0.84677 (14)	0.23813 (6)	0.56205 (5)	0.0330 (3)	
P2	0.53770 (14)	0.35222 (6)	0.65884 (5)	0.0356 (3)	
S1	0.82981 (18)	0.51569 (8)	0.57515 (8)	0.0920 (6)	
N1	0.5431 (6)	0.6134 (3)	0.51878 (19)	0.0707 (13)	
C1	0.6602 (7)	0.5719 (3)	0.5414 (2)	0.0519 (12)	
C2	1.0481 (5)	0.2237 (2)	0.61989 (17)	0.0356 (9)	
H2A	1.0149	0.2270	0.6601	0.043*	
H2B	1.0970	0.1678	0.6154	0.043*	
C3	0.1935 (5)	0.2899 (2)	0.61504 (18)	0.0400 (10)	
H3A	0.2256	0.2877	0.5746	0.048*	
H3B	0.1468	0.3458	0.6209	0.048*	
C4	0.3605 (5)	0.2739 (2)	0.66287 (17)	0.0368 (10)	
H4A	0.4067	0.2179	0.6568	0.044*	
H4B	0.3276	0.2756	0.7032	0.044*	
C5	0.9279 (5)	0.2047 (2)	0.49304 (17)	0.0350 (9)	
C6	0.9994 (6)	0.2658 (3)	0.46022 (18)	0.0483 (12)	
H6	0.9932	0.3223	0.4712	0.058*	
C7	1.0802 (7)	0.2442 (3)	0.4110 (2)	0.0641 (14)	
H7	1.1306	0.2859	0.3898	0.077*	
C8	1.0861 (7)	0.1617 (3)	0.3936 (2)	0.0651 (14)	
H8	1.1424	0.1471	0.3609	0.078*	
C9	1.0091 (6)	0.1002 (3)	0.4243 (2)	0.0588 (13)	
H9	1.0096	0.0442	0.4116	0.071*	
C10	0.9309 (5)	0.1216 (3)	0.47387 (18)	0.0449 (11)	
H10	0.8795	0.0798	0.4947	0.054*	
C11	0.6995 (5)	0.1523 (2)	0.57727 (17)	0.0343 (10)	
C12	0.5367 (6)	0.1429 (3)	0.5382 (2)	0.0523 (12)	
H12	0.5077	0.1790	0.5050	0.063*	
C13	0.4176 (6)	0.0806 (4)	0.5481 (2)	0.0721 (15)	
H13	0.3098	0.0743	0.5213	0.087*	
C14	0.4571 (7)	0.0281 (3)	0.5973 (3)	0.0702 (15)	
H14	0.3768	−0.0142	0.6037	0.084*	
C15	0.6145 (7)	0.0377 (3)	0.6370 (2)	0.0641 (14)	
H15	0.6407	0.0026	0.6709	0.077*	
C16	0.7344 (6)	0.0994 (3)	0.6269 (2)	0.0480 (11)	
H16	0.8414	0.1055	0.6542	0.058*	
C17	0.4373 (5)	0.4470 (2)	0.68533 (19)	0.0369 (10)	
C18	0.3580 (6)	0.5036 (3)	0.6417 (2)	0.0508 (12)	
H18	0.3670	0.4949	0.6009	0.061*	
C19	0.2658 (7)	0.5725 (3)	0.6579 (3)	0.0698 (15)	
H19	0.2119	0.6099	0.6280	0.084*	
C20	0.2531 (7)	0.5864 (3)	0.7180 (3)	0.0699 (16)	
H20	0.1897	0.6327	0.7290	0.084*	
C21	0.3336 (7)	0.5319 (3)	0.7613 (2)	0.0666 (14)	
H21	0.3265	0.5416	0.8021	0.080*	
C22	0.4260 (6)	0.4621 (3)	0.7455 (2)	0.0532 (12)	
H22	0.4806	0.4253	0.7756	0.064*	
C23	0.7159 (5)	0.3225 (2)	0.72144 (18)	0.0376 (10)	
C24	0.8650 (6)	0.3745 (3)	0.7347 (2)	0.0527 (12)	
H24	0.8677	0.4252	0.7136	0.063*	
C25	1.0084 (6)	0.3527 (3)	0.7782 (2)	0.0658 (15)	
H25	1.1065	0.3889	0.7866	0.079*	
C26	1.0090 (7)	0.2786 (4)	0.8094 (2)	0.0685 (15)	
H26	1.1063	0.2645	0.8392	0.082*	
C27	0.8650 (7)	0.2248 (3)	0.7963 (2)	0.0623 (14)	
H27	0.8660	0.1735	0.8168	0.075*	
C28	0.7187 (6)	0.2465 (3)	0.75300 (19)	0.0490 (11)	
H28	0.6212	0.2099	0.7448	0.059*	

### Atomic displacement parameters (Å^2^)

**Table:**

	*U*^11^	*U*^22^	*U*^33^	*U*^12^	*U*^13^	*U*^23^
Ag1	0.0491 (2)	0.03714 (18)	0.0486 (2)	0.00698 (17)	0.00730 (15)	0.00473 (16)
P1	0.0307 (6)	0.0291 (6)	0.0388 (6)	0.0003 (4)	0.0047 (5)	0.0014 (4)
P2	0.0317 (6)	0.0380 (6)	0.0375 (6)	−0.0022 (5)	0.0072 (5)	−0.0028 (5)
S1	0.0498 (9)	0.0493 (8)	0.1629 (16)	−0.0138 (7)	−0.0224 (10)	0.0306 (9)
N1	0.067 (3)	0.062 (3)	0.072 (3)	−0.004 (2)	−0.021 (2)	0.019 (2)
C1	0.060 (3)	0.039 (3)	0.051 (3)	−0.018 (2)	−0.004 (3)	0.006 (2)
C2	0.033 (2)	0.036 (2)	0.038 (2)	−0.0019 (18)	0.0040 (19)	−0.0019 (18)
C3	0.035 (2)	0.037 (2)	0.046 (3)	−0.0038 (19)	0.000 (2)	−0.0005 (19)
C4	0.032 (2)	0.033 (2)	0.045 (3)	−0.0007 (18)	0.005 (2)	−0.0008 (19)
C5	0.032 (2)	0.035 (2)	0.037 (2)	−0.0003 (18)	0.0022 (19)	0.0000 (18)
C6	0.067 (3)	0.038 (3)	0.040 (3)	−0.009 (2)	0.009 (2)	0.001 (2)
C7	0.091 (4)	0.059 (3)	0.046 (3)	−0.025 (3)	0.025 (3)	−0.006 (2)
C8	0.078 (4)	0.077 (4)	0.046 (3)	−0.013 (3)	0.028 (3)	−0.016 (3)
C9	0.078 (4)	0.048 (3)	0.053 (3)	0.001 (3)	0.019 (3)	−0.017 (2)
C10	0.051 (3)	0.038 (3)	0.047 (3)	−0.007 (2)	0.012 (2)	−0.001 (2)
C11	0.031 (2)	0.033 (2)	0.041 (3)	−0.0021 (17)	0.011 (2)	−0.0036 (18)
C12	0.043 (3)	0.059 (3)	0.052 (3)	−0.009 (2)	−0.001 (2)	0.004 (2)
C13	0.039 (3)	0.096 (4)	0.078 (4)	−0.026 (3)	0.001 (3)	−0.014 (3)
C14	0.059 (4)	0.067 (4)	0.091 (4)	−0.031 (3)	0.029 (3)	−0.007 (3)
C15	0.064 (4)	0.048 (3)	0.082 (4)	−0.013 (3)	0.016 (3)	0.021 (3)
C16	0.039 (3)	0.048 (3)	0.056 (3)	−0.006 (2)	0.006 (2)	0.012 (2)
C17	0.027 (2)	0.036 (2)	0.047 (3)	−0.0043 (18)	0.003 (2)	−0.003 (2)
C18	0.051 (3)	0.043 (3)	0.056 (3)	0.000 (2)	0.001 (2)	−0.010 (2)
C19	0.065 (4)	0.041 (3)	0.098 (4)	0.008 (3)	−0.002 (3)	0.000 (3)
C20	0.051 (3)	0.042 (3)	0.117 (5)	0.004 (2)	0.017 (3)	−0.028 (3)
C21	0.068 (4)	0.066 (3)	0.069 (4)	−0.002 (3)	0.023 (3)	−0.026 (3)
C22	0.057 (3)	0.054 (3)	0.049 (3)	0.003 (2)	0.009 (2)	−0.006 (2)
C23	0.033 (2)	0.040 (2)	0.040 (2)	0.0045 (19)	0.008 (2)	−0.0065 (19)
C24	0.044 (3)	0.053 (3)	0.058 (3)	0.001 (2)	−0.001 (2)	−0.012 (2)
C25	0.038 (3)	0.080 (4)	0.075 (4)	−0.001 (3)	−0.005 (3)	−0.029 (3)
C26	0.047 (3)	0.089 (4)	0.063 (4)	0.025 (3)	−0.009 (3)	−0.008 (3)
C27	0.057 (3)	0.069 (3)	0.060 (3)	0.021 (3)	0.007 (3)	0.011 (3)
C28	0.038 (3)	0.056 (3)	0.054 (3)	0.003 (2)	0.010 (2)	0.002 (2)

### Geometric parameters (Å, °)

**Table:**

Ag1—N1^i^	2.274 (4)	C11—C16	1.374 (5)
Ag1—P1	2.4499 (10)	C11—C12	1.388 (5)
Ag1—P2	2.5316 (11)	C12—C13	1.377 (6)
Ag1—S1	2.5726 (13)	C12—H12	0.9300
P1—C11	1.823 (4)	C13—C14	1.365 (7)
P1—C5	1.823 (4)	C13—H13	0.9300
P1—C2	1.832 (4)	C14—C15	1.363 (6)
P2—C17	1.823 (4)	C14—H14	0.9300
P2—C23	1.826 (4)	C15—C16	1.376 (5)
P2—C4	1.837 (4)	C15—H15	0.9300
S1—C1	1.633 (5)	C16—H16	0.9300
N1—C1	1.147 (5)	C17—C22	1.375 (5)
N1—Ag1^i^	2.274 (4)	C17—C18	1.380 (5)
C2—C3^ii^	1.535 (5)	C18—C19	1.374 (6)
C2—H2A	0.9700	C18—H18	0.9300
C2—H2B	0.9700	C19—C20	1.375 (7)
C3—C4	1.527 (5)	C19—H19	0.9300
C3—C2^iii^	1.535 (5)	C20—C21	1.356 (7)
C3—H3A	0.9700	C20—H20	0.9300
C3—H3B	0.9700	C21—C22	1.384 (6)
C4—H4A	0.9700	C21—H21	0.9300
C4—H4B	0.9700	C22—H22	0.9300
C5—C6	1.376 (5)	C23—C24	1.386 (5)
C5—C10	1.384 (5)	C23—C28	1.391 (5)
C6—C7	1.382 (6)	C24—C25	1.369 (6)
C6—H6	0.9300	C24—H24	0.9300
C7—C8	1.365 (6)	C25—C26	1.362 (7)
C7—H7	0.9300	C25—H25	0.9300
C8—C9	1.373 (6)	C26—C27	1.372 (7)
C8—H8	0.9300	C26—H26	0.9300
C9—C10	1.378 (5)	C27—C28	1.381 (6)
C9—H9	0.9300	C27—H27	0.9300
C10—H10	0.9300	C28—H28	0.9300
			
N1^i^—Ag1—P1	113.55 (11)	C5—C10—H10	119.6
N1^i^—Ag1—P2	109.61 (12)	C16—C11—C12	117.8 (4)
P1—Ag1—P2	102.18 (4)	C16—C11—P1	124.3 (3)
N1^i^—Ag1—S1	103.26 (10)	C12—C11—P1	117.9 (3)
P1—Ag1—S1	122.60 (4)	C13—C12—C11	120.7 (4)
P2—Ag1—S1	105.01 (5)	C13—C12—H12	119.7
C11—P1—C5	103.99 (17)	C11—C12—H12	119.7
C11—P1—C2	103.66 (17)	C14—C13—C12	120.2 (5)
C5—P1—C2	101.39 (17)	C14—C13—H13	119.9
C11—P1—Ag1	107.16 (12)	C12—C13—H13	119.9
C5—P1—Ag1	120.68 (12)	C15—C14—C13	119.9 (5)
C2—P1—Ag1	117.99 (12)	C15—C14—H14	120.0
C17—P2—C23	105.00 (18)	C13—C14—H14	120.0
C17—P2—C4	101.19 (17)	C14—C15—C16	119.9 (5)
C23—P2—C4	103.73 (18)	C14—C15—H15	120.1
C17—P2—Ag1	115.67 (13)	C16—C15—H15	120.1
C23—P2—Ag1	107.94 (13)	C11—C16—C15	121.5 (4)
C4—P2—Ag1	121.62 (13)	C11—C16—H16	119.3
C1—S1—Ag1	98.04 (15)	C15—C16—H16	119.3
C1—N1—Ag1^i^	145.6 (4)	C22—C17—C18	118.7 (4)
N1—C1—S1	177.9 (4)	C22—C17—P2	123.7 (3)
C3^ii^—C2—P1	112.6 (3)	C18—C17—P2	117.5 (3)
C3^ii^—C2—H2A	109.1	C19—C18—C17	120.7 (4)
P1—C2—H2A	109.1	C19—C18—H18	119.7
C3^ii^—C2—H2B	109.1	C17—C18—H18	119.7
P1—C2—H2B	109.1	C18—C19—C20	120.2 (5)
H2A—C2—H2B	107.8	C18—C19—H19	119.9
C4—C3—C2^iii^	110.7 (3)	C20—C19—H19	119.9
C4—C3—H3A	109.5	C21—C20—C19	119.5 (5)
C2^iii^—C3—H3A	109.5	C21—C20—H20	120.3
C4—C3—H3B	109.5	C19—C20—H20	120.3
C2^iii^—C3—H3B	109.5	C20—C21—C22	120.8 (5)
H3A—C3—H3B	108.1	C20—C21—H21	119.6
C3—C4—P2	112.1 (3)	C22—C21—H21	119.6
C3—C4—H4A	109.2	C17—C22—C21	120.2 (4)
P2—C4—H4A	109.2	C17—C22—H22	119.9
C3—C4—H4B	109.2	C21—C22—H22	119.9
P2—C4—H4B	109.2	C24—C23—C28	117.7 (4)
H4A—C4—H4B	107.9	C24—C23—P2	118.4 (3)
C6—C5—C10	118.5 (4)	C28—C23—P2	123.6 (3)
C6—C5—P1	117.4 (3)	C25—C24—C23	121.1 (5)
C10—C5—P1	124.1 (3)	C25—C24—H24	119.4
C5—C6—C7	120.7 (4)	C23—C24—H24	119.4
C5—C6—H6	119.6	C26—C25—C24	120.7 (5)
C7—C6—H6	119.7	C26—C25—H25	119.6
C8—C7—C6	120.1 (4)	C24—C25—H25	119.6
C8—C7—H7	119.9	C25—C26—C27	119.5 (5)
C6—C7—H7	119.9	C25—C26—H26	120.2
C7—C8—C9	120.0 (4)	C27—C26—H26	120.2
C7—C8—H8	120.0	C26—C27—C28	120.3 (5)
C9—C8—H8	120.0	C26—C27—H27	119.8
C8—C9—C10	119.8 (4)	C28—C27—H27	119.8
C8—C9—H9	120.1	C27—C28—C23	120.6 (4)
C10—C9—H9	120.1	C27—C28—H28	119.7
C9—C10—C5	120.8 (4)	C23—C28—H28	119.7
C9—C10—H10	119.6		
			
N1^i^—Ag1—P1—C11	−72.24 (18)	C5—P1—C11—C16	113.2 (3)
P2—Ag1—P1—C11	45.70 (13)	C2—P1—C11—C16	7.6 (4)
S1—Ag1—P1—C11	162.61 (14)	Ag1—P1—C11—C16	−117.9 (3)
N1^i^—Ag1—P1—C5	46.28 (19)	C5—P1—C11—C12	−70.8 (3)
P2—Ag1—P1—C5	164.22 (15)	C2—P1—C11—C12	−176.4 (3)
S1—Ag1—P1—C5	−78.87 (15)	Ag1—P1—C11—C12	58.1 (3)
N1^i^—Ag1—P1—C2	171.41 (18)	C16—C11—C12—C13	−2.1 (6)
P2—Ag1—P1—C2	−70.65 (14)	P1—C11—C12—C13	−178.4 (4)
S1—Ag1—P1—C2	46.26 (15)	C11—C12—C13—C14	1.1 (7)
N1^i^—Ag1—P2—C17	−71.60 (18)	C12—C13—C14—C15	0.5 (8)
P1—Ag1—P2—C17	167.68 (14)	C13—C14—C15—C16	−1.1 (8)
S1—Ag1—P2—C17	38.74 (15)	C12—C11—C16—C15	1.6 (6)
N1^i^—Ag1—P2—C23	171.17 (17)	P1—C11—C16—C15	177.6 (3)
P1—Ag1—P2—C23	50.46 (14)	C14—C15—C16—C11	0.0 (7)
S1—Ag1—P2—C23	−78.48 (14)	C23—P2—C17—C22	−32.1 (4)
N1^i^—Ag1—P2—C4	51.68 (18)	C4—P2—C17—C22	75.6 (4)
P1—Ag1—P2—C4	−69.03 (15)	Ag1—P2—C17—C22	−150.9 (3)
S1—Ag1—P2—C4	162.02 (15)	C23—P2—C17—C18	152.4 (3)
N1^i^—Ag1—S1—C1	24.7 (2)	C4—P2—C17—C18	−99.9 (3)
P1—Ag1—S1—C1	154.31 (17)	Ag1—P2—C17—C18	33.6 (4)
P2—Ag1—S1—C1	−90.17 (18)	C22—C17—C18—C19	−1.5 (6)
C11—P1—C2—C3^ii^	179.5 (3)	P2—C17—C18—C19	174.2 (3)
C5—P1—C2—C3^ii^	71.8 (3)	C17—C18—C19—C20	0.6 (7)
Ag1—P1—C2—C3^ii^	−62.3 (3)	C18—C19—C20—C21	0.6 (8)
C2^iii^—C3—C4—P2	−179.7 (3)	C19—C20—C21—C22	−0.9 (8)
C17—P2—C4—C3	69.1 (3)	C18—C17—C22—C21	1.2 (6)
C23—P2—C4—C3	177.8 (3)	P2—C17—C22—C21	−174.2 (3)
Ag1—P2—C4—C3	−60.7 (3)	C20—C21—C22—C17	0.0 (7)
C11—P1—C5—C6	161.6 (3)	C17—P2—C23—C24	−68.3 (3)
C2—P1—C5—C6	−91.1 (3)	C4—P2—C23—C24	−174.1 (3)
Ag1—P1—C5—C6	41.5 (4)	Ag1—P2—C23—C24	55.6 (3)
C11—P1—C5—C10	−22.2 (4)	C17—P2—C23—C28	118.0 (3)
C2—P1—C5—C10	85.2 (4)	C4—P2—C23—C28	12.2 (4)
Ag1—P1—C5—C10	−142.3 (3)	Ag1—P2—C23—C28	−118.1 (3)
C10—C5—C6—C7	−3.1 (6)	C28—C23—C24—C25	−1.3 (6)
P1—C5—C6—C7	173.3 (4)	P2—C23—C24—C25	−175.4 (3)
C5—C6—C7—C8	1.5 (7)	C23—C24—C25—C26	0.6 (7)
C6—C7—C8—C9	1.1 (8)	C24—C25—C26—C27	0.8 (7)
C7—C8—C9—C10	−2.1 (8)	C25—C26—C27—C28	−1.5 (7)
C8—C9—C10—C5	0.4 (7)	C26—C27—C28—C23	0.8 (7)
C6—C5—C10—C9	2.2 (6)	C24—C23—C28—C27	0.6 (6)
P1—C5—C10—C9	−174.1 (3)	P2—C23—C28—C27	174.4 (3)

Symmetry codes: (i) −*x*+1, −*y*+1, −*z*+1; (ii) *x*+1, *y*, *z*; (iii) *x*−1, *y*, *z*.
